# Acute Deep Vein Thrombosis and Pulmonary Embolism: is the Thromboaspiration Device an Appropriate Choice?

**Published:** 2020-02-20

**Authors:** C Setacci, D Benevento, G de Donato, G Galzerano, UM Bracale, F Setacci, G Palasciano

**Affiliations:** 1 Vascular Surgery University of Siena; 2 Vascular Surgery IRCSS Multimedica Milano; 3 Vascular Surgery University Federico II of Naples; Italy

**Keywords:** deep vein thrombosis, Pulmonary embolism, mechanical thrombectomy, thromboaspiration technique, devices, vein solution

## Abstract

Nowadays patients affected by deep vein thrombosis (DVT) and pulmonary embolism (PE) are studied widely but the challenge for physicians is when and how they are to be treated. Most patients present serious comorbidities that can potentially make treatment difficult. An increasing cohort of patients cannot be treated with systemic fibrinolysis but fortunately today, physicians can utilize a number of different instruments to resolve acute DVT and PE.

## I. INTRODUCTION

DVT and PE are major health problems in Europe with potentially serious outcomes in terms of mortality and morbidity, mainly because the relationship between the two pathologies is closely linked. In epidemiological studies annual incidence rates for PE range from 39–115 per 100,000 people and for DVT from 53–162 per 100,000 people.^1^

Anticoagulant drugs are recognized as effective treatments and have been introduced into clinical practice for the primary prevention of DVT and PE, for the prevention of PE in patients with a DVT diagnosis and for the secondary prevention of recurrent venous thromboembolic events over time.^2^

However, the most frequent consequence of DVT - post-thrombotic syndrome (PTS) - is not reduced by anticoagulant treatment. PTS remains the main problem in patients with previous DVT, with an incidence rate ranging from 25% to 50% in reported studies and a severe clinical manifestation characterized by ulcer development in 15% of patients.^3^

More and more authors suggest that early treatment of DVT with direct thrombolysis of the catheter, mechanical thrombolysis and drug-mechanical thrombolysis, supports the idea that recanalization of the vein, as recently evaluated in different number of studies, shows promising results in terms of improvement in quality of life and incidence reduction in PTS patients. This procedure has the advantage of preserving the venous valve function after DVT thereby preventing the development of the main physiological factors of post-DVT venous hypertension such as valve reflux and late venous obstruction.^4^ They report that particular attention should be paid to the reflux of the valve which must be prevented by operating venous thrombosed segments in which the damage induced by inflammation leads to structural valve insufficiency and in the non-involved venous segments in which venous dilation distal to obstruction leads to functional valve failure.^4^

Several studies have shown that systemic thrombolysis, surgical thrombectomy and direct catheter thrombolysis reduce PTS.^5^–^7^

New mechanical thrombus removal devices may represent safe and effective treatment for acute PE, especially in patients with absolute contraindication to thrombolysis, resulting in high technical and clinical success with such a procedure. 8–9 Moreover, coexisting DVT could be treated with thrombus removal devices thereby avoiding caval filter deployment.

Current VTE incidence is approximately 1 per 1,000 adults annually*.* The majority (almost 2/3^rds^) of patients present with DVT, while the remainder present with PE. 1-month mortality is as high as 6% for DVTs and 10% for PEs, though post-mortem studies suggest that these mortality rates are most likely underestimated as autopsy results estimate mortality to be as high as 30% suggesting that perhaps many PEs are not diagnosed at the time of death.^10^

## II. THE RATIONALE FOR AN INTERVENTIONAL APPROACH

The most alarming acute complications of proximal lower extremity DVT are PE and *phlegmasia cerulea dolens* (PCD). PCD is a rare condition in which DVT causes severe pain and swelling of the entire (lower) extremity that results in arterial circulatory compromise and acute limb ischemia from markedly increased interstitial and venous pressure. Chronic complications of proximal lower extremity DVT (recurrent VTE and post-thrombotic syndrome (PTS) are frequently debilitating. VTE recurrence is reported to be as high as 25% and patients who do not undergo anticoagulation treatment for an extended period of time have a high risk of recurrence (over 50%) within the successive 10 years. 11–12 PTS is described in up to 50% of patients within 2 years after diagnosis of symptomatic lower extremity DVT. 13

The anatomic extent of DVT has been shown to correlate with PTS incidence and severity. DVT involving the iliac and common femoral veins is more than twice as likely to cause PTS or recurrence than isolated femoro-popliteal vein thrombosis.^14^ The presence of iliofemoral DVT is also a major predictor of severe PTS. These observations have led to the so-called open vein hypothesis that relief of this outflow obstruction may prevent the development of or reduce severity of PTS.

### Catheter-based endovascular therapies

The markedly high rates of bleeding complications from systemic thrombolysis and the invasive nature of surgical approaches have led to rapid adoption of catheter-based endovascular therapies for proximal and inferior vena cava DVT over the past decade. 15

Catheter-based endovascular therapies allow thrombolytics to be directed at a specific, yet large, surface area within the thrombus (Catheter Directed Thrombolysis – CDT). Percutaneous Mechanical Thrombectomy (PMT) encompasses thrombus fragmentation, rheolytic thrombectomy with hydrodynamic catheter devices, suction thrombectomy with aspiration catheters and rotational thrombectomy. PMT can also be directly associated with CDT in some specific devices (Pharmacomechanical Cateter Directed Thrombolysis – PDCT) and in some cases angioplasty and stenting can complement and optimize the result of the thrombolysis.

Results from the recently completed Acute Venous Thrombosis study: the Thrombus Removal with Adjunctive Catheter-Directed Thrombolysis (ATTRACT) trial, a multicenter randomized control trial assessing the efficacy of pharmaco-mechanical CDT, will help provide clinicians with high-quality evidence. In patients with massive PE, systemic thrombolytic therapy has been shown to reduce mortality, decrease the risk of developing Chronic thromboembolic pulmonary hypertension (CTEPH), and improve quality of life. 16–17 A recent meta-analysis suggests that systemic thrombolytic therapy also reduces mortality in patients with sub-massive PE, but the reduction appears to be at the expense of significant major bleeding complications, including intracranial haemorrhage.^18^ These bleeding-related adverse events as well as potential treatment failure associated with systemic thrombolysis have resulted in the exploration of catheter-based thrombus removal as a major therapeutic option in these patients.^19^–^20^

## III. CURRENT GUIDELINES

Based on the available literature, the latest Italian SICVE-SIF (2016) guidelines suggest that in selected cases of acute ilio-femoral DVT, when patients are considered at a high risk for developing complications (such as PCD or venous gangrene), a CDT - PCDT or surgical thrombectomy approach followed by oral anticoagulation therapy (evaluating the risk and benefits of the treatment), is the best course of treatment. (Recommendation 12.1.9)

For pulmonary embolism in patients with absolute contraindications to thrombolysis the latest European Society of Cardiology (ESC – 2014) guidelines indicate interventional options including thrombus fragmentation with a pigtail or balloon catheter, rheolytic thrombectomy with hydrodynamic catheter devices, suction thrombectomy with aspiration catheters and rotational thrombectomy. For patients without absolute contraindications to thrombolysis however, CDT or PCDT are the preferred approaches. 21

In line with this, American ACCP guidelines (2016) recommend catheter-assisted thrombus removal over no such intervention^22^ in patients with acute PE associated with hypotension who have a high risk of bleeding, failed systemic thrombolysis, or are susceptible to shock that is likely to cause death before systemic thrombolysis can take effect (e.g., within hours) if the appropriate expertise and resources are available.^22^

## IV. ENDOVASCULAR MECHANICAL THROMBECTOMY DEVICES

Many mechanical endovascular techniques for thrombus removal have been explored over the last two decades. Starting with the simplest rotation of a Pig Tail and manual syringe suction there has been a large development of new devices in response to the clinician’s need and observation.

Some have failed to be adequately efficacious or are associated with unacceptable complication rates. Problems have included limited trackability, vessel injury and incomplete revascularization. One of the main difficulties is designing a device that can remove adequate volumes of variable age thrombus while also maintaining an acceptably small size, flexibility, and ease of use.

After a review of the literature and consulting PubMed in the English language with a series of keywords related to VTE and PMT-PCDT, we collected information on all the currently available Mechanical Thrombectomy devices. These include rheolytic thrombectomy and aspiration devices, rotational thrombectomy devices, oscillatory pharmaco-mechanical thrombectomy and aspiration devices, ultrasound-enhanced pharmacomechanical thrombectomy devices and thromboaspiration with extra-corporeal circulation devices.

The following sections describe each device, its characteristics and the current published literature on its safety and effectiveness.

### AngioJet ™ Thrombectomy System (Boston Scientific Corp)

The AngioJet ™ Thrombectomy System is a rheolityc pharmacomechanical peripheral thrombectomy device with active aspiration and pulse lytic delivery. It is designed to remove thrombus with the Venturi-Bernoulli effect having multiple, high-velocity and high-pressure saline jets which are introduced through orifices in the distal tip of the catheter to create a localized low-pressure zone resulting in a vacuum effect with ensuing engagement and dissociation of bulky thrombus.^23^

**Figure 1:**
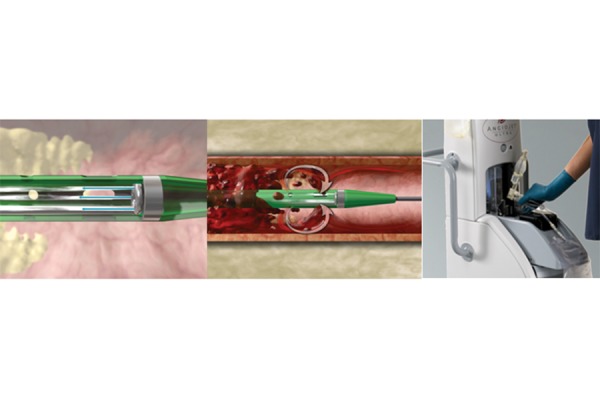
AngioJet ™ Thrombectomy System.

The system consists of a mobile console and a wide variety of catheters. The AngioJet™ console monitors and controls the system with automated self-configuration of each catheter and step-by-step procedural interface. The Console energizes the pump which sends pressurized saline to the catheter tip. Saline jets travel backwards to create a low-pressure zone causing a vacuum effect. The thrombus is sucked into the in-flow windows and the jets push the thrombus back down the catheter. The thrombus is evacuated from the body and into the collection bag. (figure 1)

Currently available catheters from 3 F to 8 F on guidewires from 0,014″ to 0,035″ allow treatment of vessels from a minimum of 1,5 mm diameter.

A total of 32 sites in the United States and Europe enrolled patients with DVT in the Peripheral Use of AngioJet™ Rheolytic Thrombectomy with a Variety of Catheter Lengths (PEARL) registry (Garcia MJ et al, 2015). 329 patients were enrolled from January 2007 through June 2013 with 67% of the patients undergoing an AngioJet procedure within 14 days of the onset of symptoms with patient characteristics and outcome data collected.

Four treatment approaches using the AngioJet ™ thrombectomy were applied: Rheolytic Therapy (RT) without lytic agent in 4% of patients, pharmacomechanical catheter-directed thrombolysis (PCDT) in 35%, PCDT and catheter-directed thrombolysis (CDT) in 52%, and RT in combination with CDT in 9%. The 3-, 6-, and 12-month freedom from rethrombosis rates were 94%, 87%, and 83%, respectively. Major bleeding events occurred in 12 patients (3.6%), none of which were related to the AngioJet ™ procedure.

Authors concluded that the PEARL registry data demonstrates that rheolytic PCDT treatment of DVT is safe and effective and can potentially reduce the need for concomitant CDT and intensive care.^24^

### Indigo ™ Percutaneous Thrombectomy System (Penumbra)

Penumbra’s Indigo System was created for cerebral vessel thromboaspiration in stroke and cerebral sinus thrombosis. With the recent addition of larger 6- and 8-F systems and a venous indication, the Penumbra Indigo® System requires a more powerful, larger-bore aspiration catheter with greater trackability to the peripheral vasculature to evacuate greater thrombus burdens from large vessels.

Indigo system’s technology helps to maintain continuous aspiration, limiting clogging of the catheter’s tip. This percutaneous system is available in four diameter options (4–8 Fr) with lengths ranging from 85 to 150 cm, allowing smaller-diameter catheters to work coaxially through larger-diameter catheters to treat long lesions in tapering vessels.

It is able to provide rapid restoration of flow to thrombosed vessels in settings in which there is inadequate time to allow thrombolysis to work. It can also be used for revascularization when thrombolytic therapy and surgery are contraindicated or prior to thrombolysis in order to decrease thrombus burden and potentially shorten lengthy infusion times. Finally, the Indigo System can be effective in removing more organized, subacute-to-chronic thrombus after thrombolytic therapy has failed.^25^

Extensive literature is available on Indigo thrombectomy for cerebral venous sinus thrombosis and for acute stroke but only a few case reports exist on Indigo Trombectomy for DVT. One of these, Vadlamudi V. (2016), reports thrombectomy with the Indigo System for a case of PCD, with a rapid restoration of the vessel patency and a solution to the symptoms, avoiding risk of amputation. He concludes that in cases of DVT when rapid debulking of thrombus burden is necessary and thrombolysis may not be feasible, thrombectomy with the Indigo System provides an efficient solution utilizing a trackable large-bore aspiration catheter and continuous vacuum technology.^26^

### AngioVac (AngioDynamics)

The AngioVac system is manufactured by Vortex Medical for AngioDynamics and is approved by The U.S. Food and Drug Administration (FDA) “to remove fresh, soft thrombi or emboli during extracorporeal bypass for up to 6 hours.” It consists of a 22F suction cannula and is combined with a veno-venous bypass circuit and a reinfusion cannula.

While on a bypass circuit, the blood passes through a filter canister which traps any undesired material such as thrombus before being reinfused into the patient via a reinfusion cannula. The tip of the suction cannula is designed with an expandable balloon funnel to optimize the aspiration and engagement area. The current design is available either with a straight or a 20 degree angle tip.

The advantage of the AngioVac over other existing devices is the large bore aspiration cannula which allows for aspiration of large volumes of thrombus.

It differs from the rheolytic devices such as AngioJet (Boston Scientific) which can cause significant hemolysis and arrhythmias. AngioVac procedures are performed under general anesthesia. Both sides of the neck and both groins should be prepped for possible access. The patient is then fully anticoagulated with heparin (ACT > 300 sec). The right internal jugular vein is the preferred access for cases of IVC thrombosis and pulmonary artery thrombosis, but common femoral vein access is preferred in patients with superior vena cava thrombus and thrombus on central venous catheters. The infusion cannula is placed in an internal jugular vein or common femoral vein which is not used for the AngioVac and where there is no thrombus. A 26F DrySeal sheath is inserted over a stiff guide wire under fluoroscopic guidance. Through this sheath, the 22F AngioVac cannula is placed over the wire. The AngioVac catheter is then slowly advanced against the thrombus and moved to and fro until the thrombus is aspirated. Special attention is needed when the cannula is advanced across IVC filters as the cannula can engage the filters and cause mangling or even displacement of the filter.^27^

Donaldson CW et al (2015) reports the first significant case series describing AngioVac use, feasibility and outcome in evacuating large caval thrombi or intracardiac masses in PE. 14 consecutive patients were retrospectivey analysed and treated with AngioVac Thrombectomies between April 2010 and July 2013 at their institution. Four patients (27%) were in shock at the start of the procedure. Peri-procedure mortality was 0% and in-hospital mortality was 13% at a mean follow-up of 23 days. No pulmonary hemorrhages, strokes or myocardial infarctions took place. Though 73% of the patients had a post procedural drop in hematocrit, only two bleeding events were related to the access site and required a transfusion. The authors concluded that AngioVac thrombectomy is feasible in critical patients with acute DVT or PE and large caval thrombi or intracardiac masses.^28^

### Aspirex (Straub Medical)

The Aspirex® System combines mechanical rotational clot fragmentation and active negative-pressure removal of thrombus particles to prevent distal embolization. It consists of a suction console operated by hand or footswitch and the catheter is available in different sizes (6–8–10 Fr). Several successful in vitro studies have been followed by the pursuit of different applications and the device has been used successfully in the treatment of acute-to-chronic thrombo-embolic arterial occlusions of the limbs, DVT, hemodialysis access grafts, bypasses, stents, pulmonary artery occlusion and inferior vena cava and aortohepatic bypass occlusions.

Eidt-Lidt G et al (2008) reported their experience with the Aspirex device on acute PE. From July 2004 to May 2007 18 patients who had met the criteria for massive PE underwent thrombus fragmentation using a pigtail catheter that was complemented in 13 patients with thrombus aspiration. The Aspirex percutaneous thrombectomy device was used in 11 of the patients. Hemodynamic, angiographic, and blood oxygenation parameters improved after the procedure. A significant increase was observed for systolic systemic BP as was a decrease in mean pulmonary artery pressure. The in-hospital major complication rate was 11.1%; one patient died from refractory shock, and one patient had an intracerebral hemorrhage (minor neurologic sequelae). No cardiovascular deaths or recurrent pulmonary thromboembolism were documented during the clinical 1-year follow-up. The group concluded that in patients with massive PE, right ventricular dysfunction and major contraindications to thrombolytic therapy, failed thrombolysis, or unavailable surgical thrombectomy, mechanical percutaneous thrombectomy appears to be a useful therapeutic alternative.^29^

### Trellis ™ Thrombectomy System (Covidien-Medtronic)

The redesigned Trellis ™system is a pharmacomechanical thrombolysis device that enables focused treatment of blood clots in cases of DVT. This latest Trellis system has been optimized to enhance drug delivery and removal of the dissolved clot. The system consists of an over-the-wire catheter with two occlusive balloons to close off the treatment area and target the drug release, an infusion zone to deliver the lytic drug and an oscillation drive unit that disperses the drug and mechanically dissolves the clot. Additionally, the new system features a larger aspiration window than the previous version which allows a better removal of the drug and the dissolved clot.

The multicenter isolated-pharmacomechanical thrombolysis device (ISOL-8, Gagne P et al, 2015) study was designed to determine the safety and efficacy of the Trellis™-8 peripheral infusion system when used as primary intervention in cases of DVT, proximal lower-extremity occlusive DVT, and to track the incidence and severity of the post-thrombotic syndrome (PTS) 2 years after treatment.

Data from patients treated with the Trellis™-8 system were retrospectively collected from six centers, including patients with occlusive lower-extremity DVT involving at least the iliac and/or common femoral vein. Patient data, procedure outcomes, complications and follow-up venous duplex and Venous Clinical Severity Scores (VCSS) were collected throughout a 24-month period.

A total of 151 limbs in 139 patients were treated. After treatment, 43.7% of the limbs showed evidence of chronic thrombus. The average amount of thrombolysis, as determined by a venogram, was highest in patients who had acute thrombus (81 ± 19.7%) compared with those who had subacute thrombus (61 ± 22.5%) and complex cases involving acute and/or subacute thrombus on chronic scars (56 ± 26.5%).

VCSS scoring showed that the number of patients with none and/or mild pain, varicose veins, and skin changes at 1-month remained stable at 12 months whereas the percentage of patients with none and/or mild venous edema improved from 71.7% at 1 month (38 of 53) to 86.8% (46 of 53) at 12 months. No clinically significant pulmonary emboli or major periprocedural bleeding events were reported. Authors concluded that patients with acute lower-extremity DVT involving the proximal veins could be safely and successfully treated with the Trellis™ System with no reports of procedural bleeding and low occurrence of severe PTS after primary treatment. 30

### Cleaner XT (Argon Medical Devices)

The Cleaner Thrombectomy System was at first indicated for the mechanical declotting of native vessel dialysis fistulae and synthetic dialysis access grafts but in 2014 Argon Medical Devices received clearance from the US Food and Drug Administration to begin marketing the CLEANER XT™ and CLEANER 15™ Rotational Thrombectomy Systems for mechanical declotting and controlled and selective infusion of physician-specified fluids, including thrombolytics, in the peripheral vasculature.

The Cleaner Thrombectomy System consists of a single sinusoidal wire design that safely macerates thrombus while preserving the vessel: a fluid vortex effectively macerates thrombus while reducing the risk of endothelial damage wall. The guidewire-like design provides good steerability and the 3-way sideport distal side hole on the catheter allows for infusion of thrombolytics and contrast media.

Koksoy C. et al (2014) reported the results of a retrospective study evaluating the use of the Cleaner Thrombectomy Device in acute or subacute VT. They analysed 41 patients with acute or subacute DVT treated with the Cleaner Thrombectomy Device between July 2012 and August 2013. The device was used in a single-session technique for patients with lower-extremity DVT.

Based on contrast venography, the extent of lysis was graded from I (< 50%) to III (complete). Sixteen patients (39.0%) had femoropopliteal thrombosis and 25 (61.0%) iliofemoral venous thrombosis. The mean duration of symptoms was 11.0 days. The mean quantity of the tissue plasminogen activator was 20.7 mg, and the mean duration of the procedure was 74.3 min. At the end of the PMT procedure, 70.7% of patients had complete (grade III) thrombus resolution. Grade I lysis were noted in one 2.4% of the patients and grade II lysis in 26.8%. Thirty-eight of the 41 patients were treated with PMT in a single session and three (7.3%) required an additional lytic infusion as a result of residual thrombi. The overall grade III, II, and I thrombus resolution rates, including the supplemental thrombolysis, were 73.2% (n = 30), 22.0% (n = 9) and 4.9% (n = 2), respectively. No mortalities occurred.

The authors concluded that the use of the Cleaner Thrombectomy Device is a promising alternative to current treatment modalities for the management of DVT in a single session.^31^

### Arrow Trerotola™ PTD® Percutaneous Thrombolytic Device (Teleflex)

The Arrow-Trerotola PTD was created for hemodialysis accesses mechanical thrombectomy but it has been also widely used for DVT thrombectomy. A mechanical thrombectomy catheter with a flexible tip and activated spinning basket, a hand-held disposable rotator drive unit and an introducer sheath (5–7 Fr) make up the system. The activated spinning basket macerates the thrombus, a catheter lumen sidearm permits catheter flushing during preparation and use, and introducer sheath and large-bore sidearm assembly simplifies thrombus removal.

Park KM et al (2014) studied patients who had undergone treatment for acute iliofemoral DVT from January 2005 to December 2011 at 2 institutions. The treatment outcomes with the Trerotola in the mechanical-thrombectomy (MT) group were compared with those obtained with Catheter-direct Thrombolysis (CDT) alone and with MT plus CDT. A total of 98 DVTs were treated. 53 with the MT and 45 with the CDT. No statistical differences were found in the clinical characteristics among the MT with CDT, MT only and CDT group. Symptom improvement was seen in 78% of the MT group, 80% of the MT with CDT group, and 71% (32 limbs) in the CDT group (P = 0.498). No difference was noted in complications during the procedures or in primary patency rates during the follow-up period.

Authors concluded that MT with the Trerotola device for acute iliofemoral DVT requires shorter procedure times and lower urokinase doses than conventional CDT while providing the same results.^32^

### Ekosonic ™ endovascular system (EKOS™ Corporation, BTG International group company)

**Figure 2:**
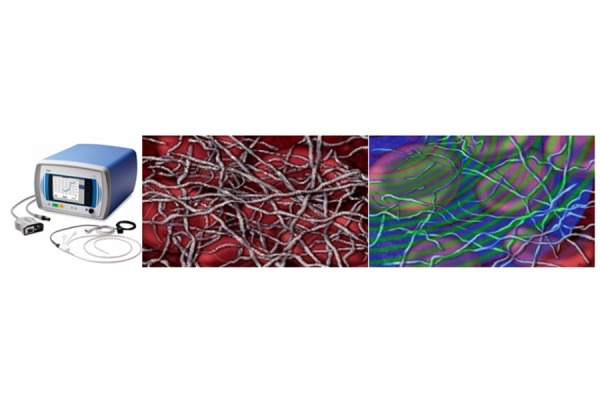
Ekosonic ™ endovascular system.

The EkoSonic™ Endovascular system with Acoustic Pulse Thrombolysis™ treatment uses targeted ultrasonic waves in combination with clot-dissolving drugs. The system uses a specific catheter and an ultrasonic core to effectively target an entire clot. The acoustic pulse field makes the fibrin more porous while creating a pressure gradient which transports the clot-dissolving drug deep inside the clot increasing clot dissolution without mechanically damaging vessels, valves or walls. At the same time the acoustic enhancement requires up to four times less drug dosage than traditional systemic delivery.^33^–^34^ (figure 2)

Zaghlool DS et al. (2016) followed a large sample population over a 5-year period to evaluate their experience with the EkoSonic endovascular system. Their aim was to prove that ultrasound-accelerated thrombolysis (UAT) provides excellent thrombolysis and midterm patency rates with minimal thrombolytic complications. Primary end points were the achievement of the complete thrombolysis. The secondary end point was to analyze the thrombolytic usage, complication rates, and midterm patency over a 1-year period^35^. A total of 48 limbs were treated with EkoSonic. Forty cases were diagnosed as acute whereas the remaining 8 were chronic. Complete thrombolysis was successful in 38 out of 48 (79%) of patients and partial thrombolysis was achieved in 10 out of 48 (21%) of patients. A total of three complications (6%) took place, all of which were minor bleeding. One-year patency was 87% with no signs of valvular reflux, comparable to published data using conventional catheter-directed thrombolysis.^35^

The clinical use of catheter directed thrombolysis with the EkoSonic Endovascular System (EKOS) in patients with acute PE had never before been evaluated. Catheter directed thrombolysis is an effective treatment modality for high risk PE patients with failed systemic thrombolysis (ST). J Sag S et al. (2016) collected thirteen consecutive patients with failed ST who had been treated with EKOS catheters and tissue plasminogen activator (t-PA) in combination with unfractionated heparin. The duration of EKOS treatment was 21.8 ± 3.8 h and the total dose of tPA was 31.2 ± 15.3 mg. One patient who presented with cardiac arrest died. The clinical status of the remaining subjects improved significantly with improvement of right ventricular functions and decrease of systolic pulmonary artery pressure without any hemorrhagic complication. None of the patients had died or suffered recurrent PE during a follow-up period of 6 months. Authors concluded that EKOS is an effective treatment modality for high risk PE patients with failed ST and can be applied with very low haemorrhagic complications.^36^

## V. CONCLUSIONS

In our preliminary analysis, simultaneous treatment of DVT and PE with the thromboaspiration devices presented herein appears to be safe and effective. Low complication rates encourage the extensive use of this technique in selected patients, however larger prospective studies are needed to assess this single treatment’s true feasibility.
